# Detecting and utilizing minority phases in heterogeneous catalysis

**DOI:** 10.1038/srep37597

**Published:** 2016-11-24

**Authors:** Urs Hartfelder, Jagdeep Singh, Johannes Haase, Maarten Nachtegaal, Daniel Grolimund, Jeroen A. van Bokhoven

**Affiliations:** 1 Institute of Chemical and Bioengineering, ETH Zurich, 8093 Zurich, Switzerland; 2 Paul Scherrer Institut, 5236 Villigen-PSI, Switzerland

## Abstract

Highly active phases in carbon monoxide oxidation are known, however they are transient in nature. Here, we determined for the first time the structure of such a highly active phase on platinum nanoparticles in an actual reactor. Unlike generally assumed, the surface of this phase is virtually free of adsorbates and co-exists with carbon-monoxide covered and surface oxidized platinum. Understanding the relation between gas composition and catalyst structure at all times and locations within a reactor enabled the rational design of a reactor concept, which maximizes the amount of the highly active phase and minimizes the amount of platinum needed.

Heterogeneous catalysis is the key technology for energy conversion and storage in the refinery and in the production of chemicals. It enables an efficient use of energy and feedstock in large-scale reactions, while keeping the amount of waste to a minimum. The rational design of catalysts requires an in-depth understanding of the catalyst structure on the molecular level and in particular an understanding of how different structures contribute to activity^1^. O*perando* spectroscopic techniques combined with theoretical calculations are generally applied to determine the structure of functioning catalysts in actual reactors under catalytic conditions^2^,^3^,^4^,^5^,^6^,^7^,^8^. However, the relation between the structure of a catalyst measured *operando* and its performance is no guarantee that the active phase or the catalytically active site is identified^9^.

Because of conversion of the feed, the conditions inside a reactor and, thus, the catalyst structure may change along the direction of flow with an accompanying change in catalytic reactivity^10^,^11^. Overall, these factors complicate the unambiguous identification of the origin of catalytic reactivity. More recently transient methods are employed to identify the active phases and to differentiate between spectator species and active species^12^,^13^. Transient spectroscopy combined with mass spectrometry is a particularly powerful tool, since it combines structural information at a single point in the reactor with integral activity information. This enables the quantitative description of structure and activity throughout the reactor.

The oxidation of carbon monoxide over platinum-group metals is a topic of many studies in heterogeneous catalysis, due to its practical application and as a model reaction^14^. Despite its apparent simplicity, this reaction is remarkably complex, with different kinetic regimes, light-off, and oscillating kinetics^15^,^16^,^17^,^18^,^19^. Oxidation of carbon monoxide over coinage metals occurs in three regimes: (i) a carbon monoxide-inhibited low temperature regime where the reaction rate is determined by carbon monoxide desorption, (ii) a transition regime with so far not well characterized structure and activity, and (iii) a high-temperature regime where the rate of carbon dioxide formation is either limited by mass-transfer on a metallic surface or by the lower reactivity of the oxidized surface. There is still no quantitative assessment as to which phase contributes to conversion in an actual reactor under catalytic conditions.

Time-resolved x-ray absorption spectroscopy (XAS) is a powerful tool to investigate heterogeneous catalysts under reaction conditions^20^,^21^,^22^. It has been applied to study carbon monoxide oxidation on platinum catalysts^23^, and is especially useful, because spectral features in the Pt L_3_ edge enable distinguishing between oxidized, bare, and oxygen or carbon monoxide-covered platinum nano-particles^24^. Today’s high time resolution makes it possible to measure structural changes on a sub-second time scale and to relate structural changes to kinetics^25^. Our work deals with the application of transient spectroscopy combined with mass spectrometry and chemometric analysis to identify the various catalytic phases throughout the reactor. We characterized the structure and time-resolved conversion of carbon monoxide over the catalyst during light-off and at steady state and were thus able to identify that multiple structures are present during reaction. Subsequently, we designed a reactor to facilitate the creation of a highly active region and thus minimize the amount of coinage metal needed.

## Results and Discussion

**Figure 1:**
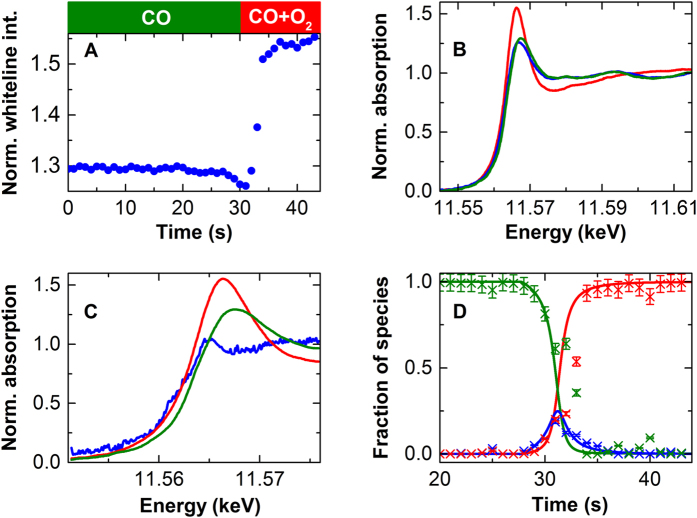
There are structural changes in a 2 wt% platinum on Al_2_O_3_ catalyst during the switch from carbon monoxide to the catalytic mixture of carbon monoxide and oxygen as observed by transient quick XANES and EXAFS at the Pt L_3_ edge. (**A**) Whiteline intensity of normalized Pt L_3_ XANES spectra versus time during the switch indicates that right after the switch, starting from a carbon monoxide-poisoned surface, a short-lived phase forms, as indicated by a temporary decrease in whiteline intensity followed by a rapid increase. This rapid increase is indicative of the formation of a surface oxide^36^. (**B**) Corresponding XANES spectra at 0 s (green), 31 s (blue), and 43 s (red). (**C**) Spectra of the carbon monoxide-covered platinum (green), surface platinum oxide (red) and the intermediate (blue) determined by the genetic algorithm procedure. (**D**) Fractions of the surface occupied by carbon monoxide (green), the intermediate species (blue) and a surface oxide (red) as determined by linear combination fitting of the spectra in (**C**) (crosses) as a function of time. The estimated error was 5% based on a systematic analysis of the sensitivity of the fit towards variation in relative amounts of each component.

Figure 1A illustrates the change in maximum whiteline intensity of the individual XAS spectra versus time, which is indicative of platinum nano-particles changing their structure upon rapid switching of the gas phase from carbon monoxide to a carbon monoxide/oxygen mix (1:1), which induces the catalytic reaction. The whiteline intensity increased as expected for the transition from a carbon monoxide covered, reduced platinum surface to a surface oxide as determined by EXAFS analysis^26^. However, before the increase, a small decrease in the whiteline intensity occurred. This identifies the existence of a third phase during the transition, which is confirmed by principal component analysis of the full XANES dataset (Figure S2, details in the SI). Principle component analysis is a well-established tool to quantify all existing components in a XAS spectrum^27^,^28^. It enables a quantitative view of the entire data set not provided by whiteline intensities or areas. The maximum whiteline intensity was used here for illustrative purposes. In addition to the increased whiteline intensity in the spectra recorded beyond 30 s, there is a small shift towards lower edge energies. This shift is the result of the removal of carbon monoxide and the associated back bonding interaction^24^,^29^.

Figure 1C shows the XANES spectra of the initial and the equilibrium states as well as the spectrum of the third (intermediate) component obtained by optimizing the linear combination fit of the data with a genetic algorithm. The obtained spectrum closely resembles that obtained for metallic platinum nanoparticles free of adsorbates and supported on alumina^24^ and differs from that representative of a surface with chemisorbed oxygen^30^,^31^,^32^, which shows enhanced whiteline intensity without edge shift. Chemisorbed oxygen atoms are highly reactive, making them short-lived and therefore appear in low concentrations only (vide infra), too low to induce changes in the spectra. We choose to describe this lowly covered surface as a distinct intermediate species, since it behaves as one spectroscopically and results in distinct catalytic behavior. Activity of this intermediate phase is high, because it enables carbon monoxide and dissociated oxygen to react in a Langmuir-Hinshelwood type mechanism.

We repeated the time-resolved XAS measurement at different temperatures and the obtained spectrum of the intermediate was virtually identical in all cases, thus confirming the structure (Figure S3). Figure 1D represents the time-dependence of the experimentally determined three species, as expected for a reaction mechanism with an intermediate. The lines in the figure indicate the results of fitting a kinetic reactor model (vide infra) to the data from the linear combination fit. The model takes into account the concentration of the gas phase and gives a good fit of the fractions of each phase; in particular, the initially slow, then faster decrease in the concentration of the carbon monoxide-covered platinum is reproduced.

**Figure 2:**
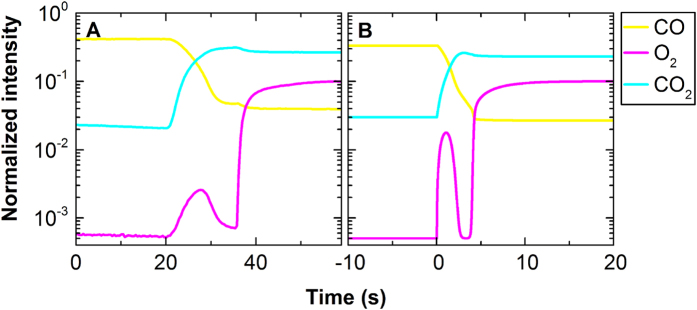
(**A**) Measured and (**B**) simulated mass spectrometry traces of carbon monoxide, oxygen, and carbon dioxide during the switch from a carbon monoxide atmosphere to a CO/O_2_ atmosphere. Measured and simulated data have identical features, including a pronounced peak in the oxygen signal before the increase to equilibrium level, caused by the lower activity of the initially present carbon monoxide-poisoned nanoparticles. There also is an overshoot in the carbon dioxide signal at the moment of light off, originating from the reaction of the chemisorbed carbon monoxide on the platinum nanoparticles, as observed earlier in oscillatory kinetics^11^.

Figure 2A depicts the composition of the reactor exhaust as determined by mass spectrometry during the switch. The most notable features are: the carbon dioxide signal increases and the oxygen signal goes through a maximum. The carbon dioxide signal reaches a maximum just before stabilizing at equilibrium. These features in the exhaust gas composition are clearly reproduced by the reactor simulation, which is based solely on the kinetic model that describes the experimentally determined structures using the XAS data (Fig. 2B). Because the mass spectrometry data and the structural characterization are simultaneously performed, they can be quantitatively correlated.

**Figure 3:**
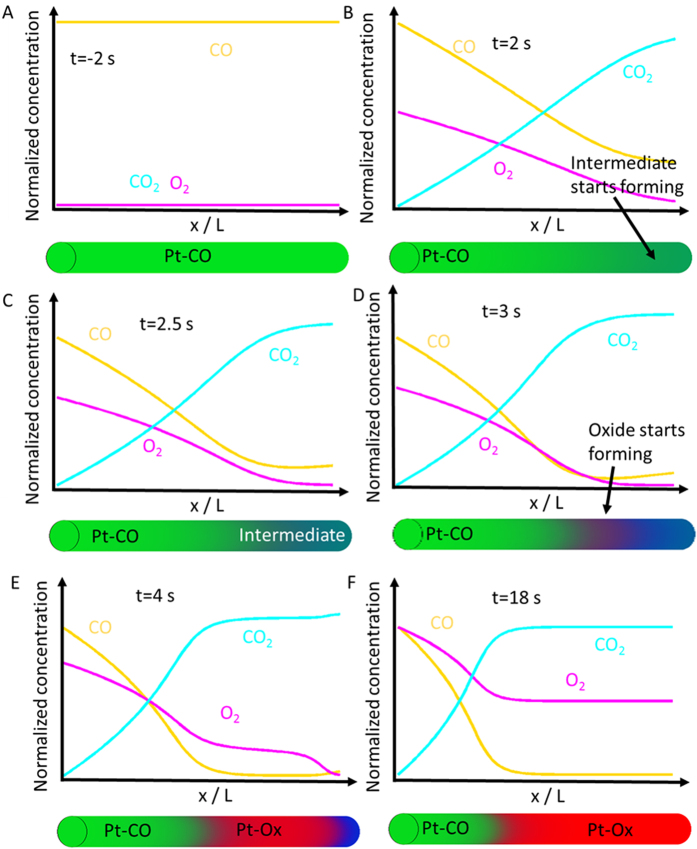
Simulated gas phase concentrations (yellow: carbon monoxide; magenta: oxygen; cyan: carbon dioxide) and structure of the nanoparticles throughout the reactor obtained from the kinetic reactor model, as depicted below each of the graphs: chemisorbed carbon monoxide (green), surface oxide (red) and surface intermediate (dark blue) at t = −2 s (**A**), 2 s (**B**), 2.5 s (**C**), 3 s (**D**), 4 s (**E**) and 18 s (**F**). After the switch to catalytic conditions, carbon dioxide production starts, leading to a region near the outlet of the reactor where both carbon monoxide and oxygen are depleted and, thus, a significant amount of the surface shows a very low coverage (intermediate phase, **B**–**F**). With increasing oxygen concentration that surface gets oxidized, roughly in the middle of the reactor (**D**) and progressing downstream (**E**,**F**).

Figure 3 illustrates how surface structure and gas phase composition influence each other throughout the reactor at different snapshots in time. The catalyst structures are based on the XAS data (Fig. 1); the mass spectrometry data are modelled with finite element simulations using mechanistically established assumptions (Langmuir-type reaction mechanism including competitive adsorption and reaction and a fast desorption step) and a simplified mass-transfer-model (for details on the modelling approach, see the SI). The kinetic mechanistic model assumes that adsorbed carbon monoxide prevents oxygen from reacting with the surface. Desorption of carbon monoxide frees a site that enables oxygen activation. Surface oxygen may react with co-adsorbed carbon monoxide freeing sites for oxygen to adsorb and react either to carbon monoxide or to platinum to form a surface platinum oxide. This surface can reduce by reaction with surface carbon monoxide. Before the switch (Fig. 3A), the catalyst is carbon monoxide covered throughout the reactor, as the only reactant in the gas phase is carbon monoxide. Two seconds after the switch to catalytic conditions (Fig. 3B), carbon monoxide oxidation has started. This leads to a gradual increase in the carbon dioxide signal at the reactor exhaust and a decrease of oxygen and carbon monoxide further into the reactor. A surface covered in carbon monoxide is active after desorption of carbon monoxide, so that free surface is available for oxygen to dissociatively chemisorb and to react to adsorbed carbon monoxide^33^. Carbon monoxide and oxygen are depleted in the downstream parts of the reactor, resulting in decreased carbon monoxide coverage (Fig. 3C), especially near the outlet of the reactor where the concentrations are lowest. Not all oxygen is converted 2.5 s after the switch and is thus present for a short period after the switch in the exhaust of the catalyst, yielding the local maximum in concentration (Fig. 2). The appearance of this oxygen peak depends on the oxygen to carbon monoxide ratio and the total gas flow.

As the concentration of oxygen surpasses the amount required to oxidize all the carbon monoxide (at the catalyst surface), the intermediate surface becomes rapidly oxidized (Fig. 3D). This occurs first in the middle of the reactor, where the gas phase oxygen interacts with a surface virtually free of carbon monoxide and where the oxygen concentration is significant. This surface oxidation removes all the oxygen from the feed, which accordingly is no longer detected in the mass spectrometer. The higher oxygen concentration leads to a faster reaction near the inlet of the reactor; thus, there is faster depletion of carbon monoxide resulting in a shift of the carbon monoxide covered surface towards the front end of the reactor. At the same time, the oxidized zone expands downstream to the point where all gas phase oxygen is used up (Fig. 3E). At steady state, the surface of the catalyst near the inlet of the reactor is covered by a significant amount of carbon monoxide, whereas downstream the surface is fully oxidized. This overall behavior is determined by the local concentration ratio of carbon monoxide and oxygen, which goes from 1:0 to 1:1. Since the stoichiometric ratio is 2:1, the reactor goes from an oxygen-poor to an oxygen rich state.

**Figure 4:**
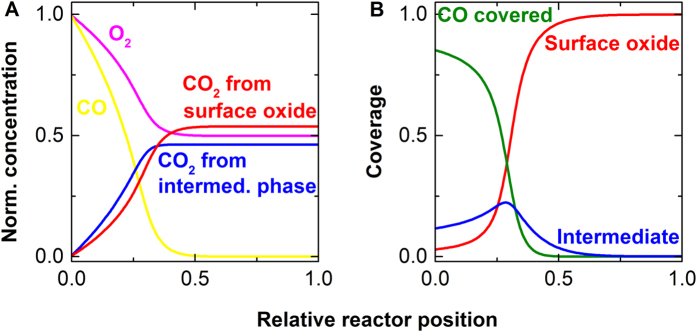
(**A**) The simulated production of carbon dioxide over each of the active phases (**B**) and the structure of the catalyst at the steady-state in the catalytic reactor with a catalytic feed of carbon monoxide and oxygen. Close to the inlet of the reactor, activity is dominated by reaction of oxygen with surface freed by carbon monoxide desorption. The desorption of carbon monoxide is rate limiting^32^. As carbon monoxide coverage decreases, surface oxide forms, contributing to the reaction in concert with the intermediate phase that contributes most at the lowest concentration of carbon monoxide. Overall, highest activity is achieved in a relatively small area of the reactor close to but not directly at the inlet.

The transition area between these two phases contains only a small amount of platinum nanoparticles with very low surface coverage of reactants, but this phase contributes significantly to the conversion of carbon monoxide (Fig. 4). This is the only phase in which both oxygen and carbon monoxide are free to adsorb and react, and it thus represents the most active phase possible^34^.

Figure 4A shows the modelled concentrations of gas phase species throughout the reactor at steady-state conditions obtained from the kinetic reactor model. Near the reactor inlet, desorption of carbon monoxide enables oxygen activation and formation of carbon dioxide, which creates the conditions under which the intermediate phase and then surface oxide form.

The yield over each of these two structures is almost the same (Fig. 4A) even though the intermediate phase makes up only a small fraction (Fig. 4B), and the region in which conversion occurs is tightly confined in the reactor at the interface between the carbon monoxide covered and the oxidic region. This matches previous observations suggesting that the transition region between these two phases may be crucial for catalytic activity^33^. Based on the experimental and simulation results, we propose the following explanation for light-off phenomena in carbon monoxide oxidation, which follows the position within the catalyst bed: In the low temperature regime, carbon monoxide oxidation proceeds according to the Langmuir-Hinshelwood mechanism over reduced platinum, with the rate of reaction increasing with temperature and with decreasing carbon monoxide pressure. When operating in excess oxygen, this leads to larger oxygen to carbon monoxide ratios towards the outlet of the reactor. The carbon monoxide coverage decreases, which creates a very reactive surface with a low steady state coverage in carbon monoxide and oxygen. At sufficiently low carbon monoxide coverage, more oxygen dissociatively adsorbs than can be immediately converted, producing the rapid increase in activity. During light-off, this transition zone propagates towards the inlet side of the reactor where it is stabilized, and some amount of reduced, carbon monoxide covered platinum is maintained at the reactor entrance (enforced by the local carbon monoxide to oxygen ratio). The structure of the intermediate phase and its reactivity has not been solved in previous XAS studies^11^,^35^,^36^,^37^. Measuring the catalyst structure space-resolved using a widened beam and measuring the Pt L_3_ edge spectra using a space-resolved detector identified the existence of at least two different structures of platinum under steady state catalytic conditions (Figure S4 shows the experimental setup; Figure S5 illustrates the changing structure of platinum along the catalytic reactor). Platinum is fully reduced and covered with carbon monoxide at the reactor inlet; at about 40% of the length of the catalyst bed, the structure changes into an oxidized one, consistent to the one predicted from the transient experiment. Increasing the oxygen content shifted the transition zone towards the outlet of the reactor, which is predicted by model that describes the catalyst structure within the reactor (Fig. 3). The energy resolution (1 eV) and data quality at the chosen measurement conditions were insufficient to identify the intermediate phase, indicating the superiority of the transient experiment in terms of sensitivity in a given measurement time.

**Figure 5:**
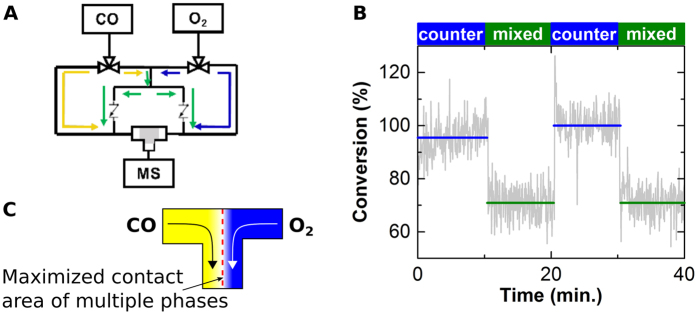
(**A**) Illustration of a switchable mixed/counter flow setup to test a T-shaped catalyst bed (yellow: carbon monoxide, blue: oxygen, green: mix); gas flow as indicated by the green arrows gives a feed of mixed carbon monoxide and oxygen; the yellow and blue arrows, respectively, indicate carbon monoxide and oxygen flowing from different sides into the catalyst bed, resulting in an interface between the two gases within the catalyst bed, (**B**) Conversion efficiency of CO_2_ measured via mass spectrometry at the outlet of the reactor as a function of time during the switches between the two flow modes. Counter flow of oxygen and carbon monoxide creates a transition zone, thereby increasing the amount of the highly active intermediate phase and, thus, activity, and (**C**) illustration how a counter flow of oxygen and carbon monoxide in the T-shaped reactor maximizes the region where multiple phases are present in the reactor, enhancing the fraction of most active catalyst boosting the conversion.

Since most conversion occurs in the transition zone, which is located close to the inlet, there is a very inefficient use of platinum, with large oxidic and carbon monoxide poisoned fractions mostly unused. However, the length of the reactor is crucial for initiating thermal light-off, since it determines at what temperature a sufficiently high oxygen to carbon monoxide ratio is first reached to induce ignition at a given ratio in the feed. Using less catalyst requires a higher activity to reach conditions where the carbon monoxide surface coverage is low enough for oxygen to freely adsorb and react to adsorbed carbon monoxide, auto-catalytically removing carbon monoxide to very low steady state coverage. An alternate reaction scheme aimed at producing a larger transition zone between carbon monoxide covered and oxidic platinum avoids this situation and potentially achieves high conversion with less platinum and/or at lower temperatures. Figure 5A presents an example of a flow setup that exploits a T-shaped reactor geometry with two separate inlets, enabling the switch between a mixture of oxygen and carbon monoxide (essentially identical to a conventional plug-flow reactor) and having carbon monoxide and oxygen in counter flow, with the goal of increasing such an active transition zone. Figure 5B shows that in the case of counter flow of oxygen and carbon monoxide, the same amount of catalyst yields much higher conversion than with the mixed flow. An estimated 70% conversion occurred when flowing mixed carbon monoxide and oxygen while full conversion was achieved in counter-flow mode. This is the result of lower local carbon monoxide to oxygen ratios in parts of the reactor, inducing high activity locally. Figure 5C shows how a counter flow of oxygen and carbon monoxide increases the area within the reactor where multiple catalyst phases co-exist. As in the classical plug-flow reactor, there is a carbon monoxide covered zone (right side) and an oxidic one (left side). At their interface, there is mixed phases present, which leads to the highly active phase. In the T-reactor, the existence of the mixed zone is enforced by the gas flows and reactor geometry. Unlike in the plug-flow reactor, the mixed phases are present at all temperatures, affording the higher conversion. This T-reactor approach is a similar to, but distinct from the use of membrane reactors as well as dosing schemes or multiple axial injections^38^ for selectivity enhancement. Those schemes aim at controlling the local gas concentrations, to control the reaction selectivity, typically to prevent overreaction and full oxidation. The concept in our reactor is a first step towards maximizing the concentration of a highly active phase, which yields a much more active system.

## Conclusions

In conclusion, the local composition of the gas phase, the local catalyst structure, and activity are intricately linked in heterogeneous catalysis. Transient spectroscopy using QEXAFS with sub second resolution combined with reactor modelling enable the quantitative assessment of their relationships. The formation of a highly active region was identified using this approach. This region, that includes some surface free of adsorbates, which enables carbon monoxide and oxygen to rapidly react, contributes to the catalytic activity. The fraction of free surface observed in this region is both the result of rapid reaction and the cause for it through its capability to adsorb oxygen. Rapid oxidation of carbon monoxide or of the metal surface occurs. Only transient methods and reactor modelling are able to capture and quantify such catalytically active surfaces. The use of the catalyst is very inefficient; good reactor design facilitates the emergence of the most active region and, thus, high conversion. This decreases the required amount of catalyst needed to achieve performance.

## Methods

### Catalyst preparation and characterization

The Pt/Al_2_O_3_ catalyst for the x-ray absorption experiment was prepared by incipient wetness impregnation of tetra amine platinum nitrate on aluminum oxide powder to give a metal loading of 2 wt%^26^. The catalyst was subsequently dried, calcined and reduced to give platinum nanoparticles supported on alumina. Transmission electron microscopy confirmed that the platinum particles had a narrow size distribution with an average particle size of 0.9 nm^26^. Since the material used for the experiments described here was produced in the same batch as that in ref. 26 the detailed results of the characterization are not repeated here.

The catalyst for the T-reactor tests and the space-resolved XAS experiment was synthesized by incipient wetness impregnation. 20 mg of tetra amine platinum nitrate (Aldrich) were dissolved in 1.85 mL deionized water. The resulting solution was added dropwise to 2 g of aluminum oxide with constant mixing for a nominal metal loading of 1 wt%. The resulting mixture was calcined under air at 500 °C for 5 h, then reduced in a flow of 100 mL/min of 5% H_2_/He for 2 h at 250 °C. The resulting particles had a mean particle size of 1.9 nm. A typical TEM micrograph and a histogram of the size distribution are shown in Figure S1.

### XAS experiments

For the x-ray absorption experiment, twenty-two milligrams of the catalyst were loaded into an x-ray absorption cell with plug flow geometry, mounted vertically^38^,^39^. The catalyst was reduced in a flow of 4% hydrogen in helium at 200 °C. The switch was carried out by passing 27.3 mL/min of 10% carbon monoxide in He through the cell at 245 °C, and then adding an additional flow of 2.7 mL/min oxygen to give a 1:1 ratio of carbon monoxide to oxygen. The reactor was then run at a space velocity of 25 s^−1^, ensuring that the time scale of the switch is much faster than the measurement.

QEXAFS data were recorded at the SuperXAS (X10DA) beam line of the Swiss Light Source in Villigen, Switzerland^25^. The polychromatic radiation was monochromatized with a channel cut Si(111) crystal in the QEXAFS monochromator. The spectra were recorded in transmission geometry, using ion chambers for detection, with a reference Pt foil mounted after the sample for absolute energy calibration. Spectra were measured in the middle of the catalyst bed, where it was previously shown that oxidation and reduction occurred depending on the composition of the gas phase^11^, and with a spot size of 1,000 × 100 μm^2^ (H x V).

The T-shaped reactor bed was created by filling a stainless steel Swagelok T-piece with catalyst powder and keeping it in position with quartz wool plugs. The catalyst was exposed to a flow of 1 mL/min carbon monoxide, 0.5 mL/min oxygen, and 18.5 mL/min helium (the helium flow was added to the branches in such a way that each branch had a total flow of 10 mL/min). The reactor was heated through the use of a heat blower. The temperature was calibrated after the measurement, by positioning a thermocouple at the place of the sample in the reactor bed. Switching experiments were carried out at a reactor temperature of 225 °C.

## Additional Information

**How to cite this article**: Hartfelder, U. *et al*. Detecting and utilizing minority phases in heterogeneous catalysis. *Sci. Rep.* **6**, 37597; doi: 10.1038/srep37597 (2016).

**Publisher’s note:** Springer Nature remains neutral with regard to jurisdictional claims in published maps and institutional affiliations.

## Supplementary Material

Supplementary Information
