# Supplementary material for: Detecting and utilizing minority phases in heterogeneous catalysis
Source: Sci Rep. 2016 Nov 24;6:37597. doi: 10.1038/srep37597 (PMC5121607; doi:10.1038/srep37597)
Supplement: Supplementary Information [file srep37597-s1.pdf]

Supporting Information for:

Detecting and utilizing minority phases in heterogeneous catalysis

Urs Hartfelder[1], Jagdeep Singh[1], Johannes Haase[2], Maarten Nachtegaal[2], Daniel Grolimund[2], Jeroen A. van Bokhoven[1,2,*]

[1] Institute of Chemical and Bioengineering, ETH Zurich, 8093 Zurich, Switzerland

[2] Paul Scherrer Institut, 5236 Villigen-PSI, Switzerland

[*] jeroen.vanbokhoven@chem.ethz.ch

Catalyst preparation and characterization

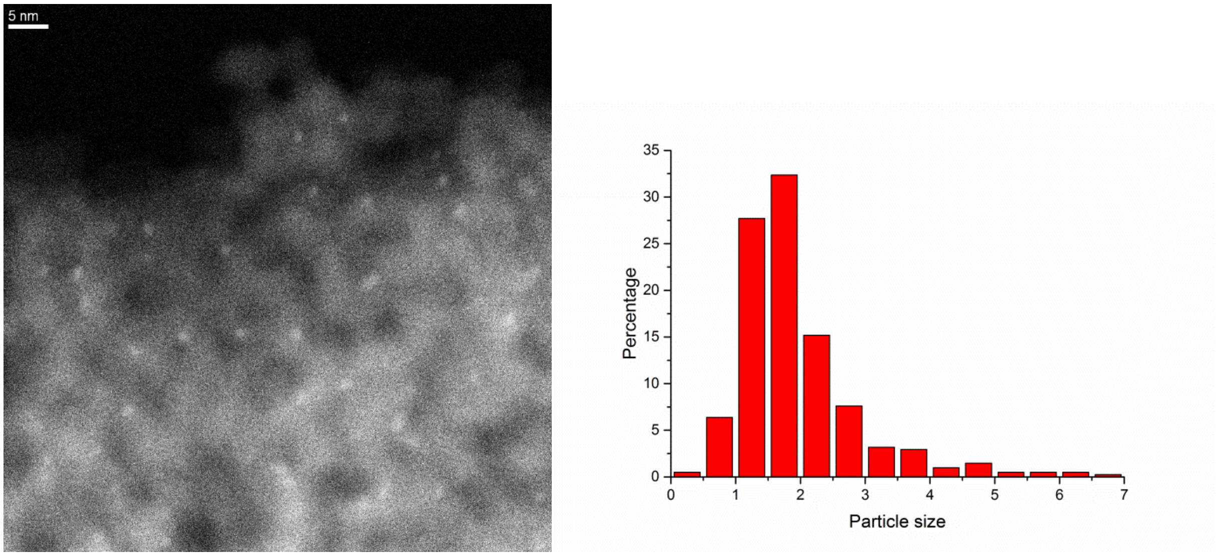

Fig. S1: A typical TEM micrograph and the histogram of the particle size distribution of the catalyst used in the T-reactor experiment.

XAS data treatment

Principal component analysis (PCA) is a widely used technique in data analysis which transforms a data set into so called principal components to simplify the description of complex data sets and to recognize patterns that are otherwise difficult to discern. It is widely applied in x-ray absorption spectroscopy[1,2]. The principal component analysis of the full XANES data presented in Figure 1 yielded two significant principal components. The first PC describes 97.3% of the variance in the data set, the second PC 1.3% and all further components less than 0.3%. Figure S2 shows the spectra plotted in the space spanned by

the first two principal components. The spectra from 0 to 29 s and the spectra from 34 to 44 s each form a group clearly separated along the first PC, but spanning the same range in the second PC. The spectra from 30 to 33 s appear in this order along the first PC, thus showing the transition from carbon monoxide to oxygen. However, they are also separated from both the initial and the final spectra in the second PC, which is indicative of a transient species. Therefore, the three species suggested by the number of principal components can be identified as carbon monoxide covered platinum, oxidic platinum and an intermediate species with an unknown spectrum. This does not exclude the possibility of additional intermediates, but they may be too short-lived and thus be present in too low concentrations to be observable with the time resolution and sensitivity in this experiment.

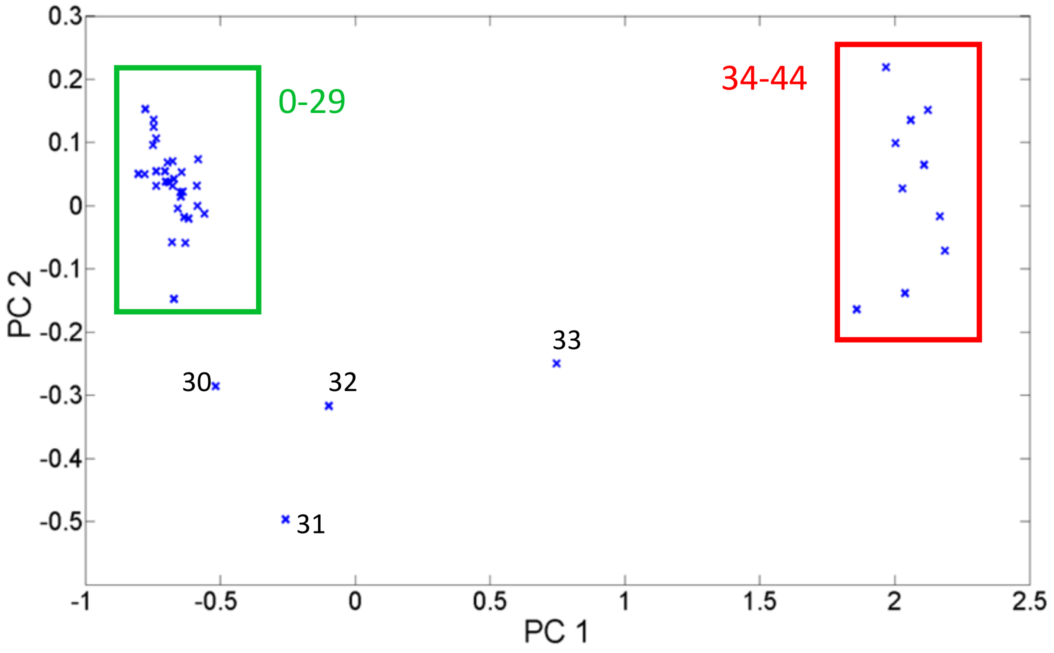

Fig. S2: The original spectra plotted in the space spanned by the first two principal components, with the numbers indicating the time of measurement. The first principal component distinguishes CO-covered and oxidic platinum, whereas the second principal component indicates the presence or absence of an intermediate species.

The spectra of two of the three species present in the measured samples are known. Isolating the spectrum of the intermediate is however made more difficult due to the fact that its concentration is unknown at each point in time. Under the assumption that the measured spectrum at each time is a linear combination of the spectra of the three species (usually a valid assumption for XANES spectra, as long as the number of phases is known[1,2]), this problem can be treated as an optimization problem: the goal is finding the intermediate spectrum that is best able reproduce the measured spectrum at each time as a linear combination with the two known spectra. An efficient approach to optimization problems are genetic

algorithms, which seek better solutions through random variation and selection based on a fitness function.[3]

The genetic algorithm (GA) used to carry out that optimization was implemented using the optimization toolbox of Matlab 7.12[4] as previously described[5]. The fitness function computes the optimal linear combination of three spectra to reproduce the measured spectrum at four selected times (30-33 s after the start of the experiment, where the concentration of the intermediate was expected to be highest). The three spectra used for this calculation are the average of the first five spectra, the average of the last three spectra, and a candidate solution from the population of the genetic algorithm. The fitness value of the candidate solution is the sum of the squared residual of the linear combination fit at the selected times. In order to enforce the shape of a normalized X-ray absorption spectrum, a minimum value for the contribution of the candidate solution in the linear combination was set between 2% and 10%. In addition, the initial population was seeded with tangent functions approximating the shape of the measured spectra. The genetic algorithm was run for 15000 generations, at which point the fitness function was not improving significantly anymore. The spectrum obtained from the genetic algorithm was then used to calculate the concentration of each species for every measured spectrum through a linear combination fit using MatLab.

Reactor simulation

The kinetic constants of the reactor model were found by optimizing the kinetic constants in the differential equations describing adsorption, desorption, and reaction (eq. S1-S7) to fit the surface concentrations obtained from the linear combination fit of the XAS spectra for the middle of the reactor. The simulation was carried out using 20 points in space and 10000 points in time, corresponding to a spatial resolution of 0.25 mm and a time resolution of 2 ms. For plotting, the results were extrapolated by a factor of 10. Two additional constants were introduced, the offset between the measured and simulated times, and a constant describing the shape of the oxygen increase after the switch.

$$\frac{d}{dt}CO = -k_1\, c_{CO}\, \theta_{free} + k_2\, \theta_{CO} - k_7\, c_{CO}\, \theta_{Ox} \tag{S1}$$

$$\frac{d}{dt}\theta_{CO} = k_1\, c_{CO}\, \theta_{free} - k_2\, \theta_{CO} - k_5\, \theta_{CO}\, \theta_O \tag{S2}$$

$$\frac{d}{dt}O_2 = -k_3\, c_{O_2}\, {\theta_{free}}^2 + 0.5\, k_4\, {\theta_O}^2 \tag{S3}$$

$$\frac{d}{dt}\theta_O = k_3\, c_{O_2}\, {\theta_{free}}^2 - 0.5\, k_4\, {\theta_O}^2 - k_5\, \theta_{CO}\, \theta_O - k_6\, \theta_O \tag{S4}$$

$$\frac{d}{dt}\theta_{Ox} = k_6\,\theta_O - k_7\,c_{CO}\,\theta_{Ox} \tag{S5}$$

$$\frac{d}{dt}\theta_{free} = -k_1\,c_{CO}\,\theta_{free} + k_2\,\theta_{CO} + k_3\,c_{O_2}\,\theta_{free}^{\;2} - 0.5\,k_4\,\theta_O^{\;2} + 2\,k_5\,\theta_{CO}\,\theta_O + k_7\,c_{CO}\,\theta_{Ox} \tag{S6}$$

$$\frac{d}{dt}CO_2 = k_5\,\theta_{CO}\,\theta_O + k_7\,c_{CO}\,\theta_{Ox} \tag{S7}$$

Space-resolved XAS

Due to the presence of concentration gradients through a fixed-bed reactor, it is often desirable to measure catalyst structure as a function of position in the reactor. This can be achieved by scanning a microfocused beam along the reactor, or by using a detector that provides spatial resolution together with a widened x-ray beam. The latter method, which is applied in this work, is roughly analogous to optical microscopy, in the sense that the sample is illuminated and the transmitted light hits a space-resolved detector, typically a CCD camera[6Fehler! Textmarke nicht definiert.]. By varying the incident photon energy, a XAS spectrum can be recorded for each pixel in the detector.

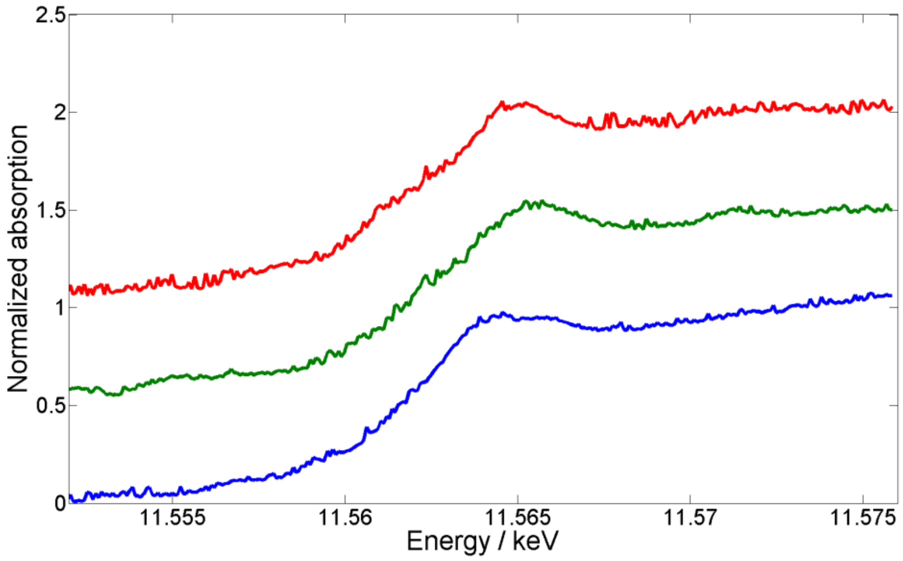

Fig. S3: Spectra obtained by the genetic algorithm from the datasets measured at 250 °C (blue), 268 °C (green) and 295 °C (red). The spectra show a large degree of consistency from independent runs of the analysis procedure.

To compensate for variations in beam intensity across the detector, background images (i.e. without any sample in the beam path) are also measured. Since the intensity distribution might also fluctuate in time, this was done at each energy step, necessitating repeated movement of the sample stage.

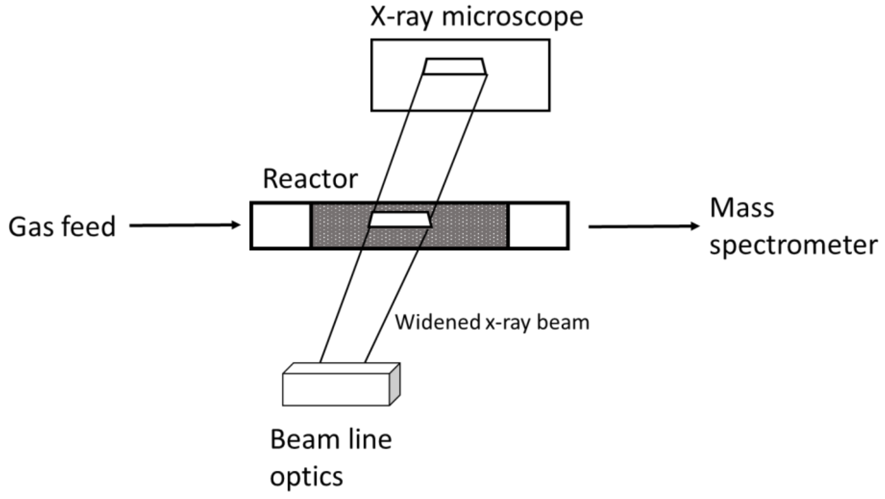

Fig. S4: Scheme of the setup for space resolved XAS experiments. The capillary reactor is mounted on a movable sample stage including the heater. The gas supply is controlled by thermal mass flow controllers. Exhaust gas composition is monitored by mass spectrometry. Monochromatized high energy x-rays are provided by the beam line optics. The images are recorded on the x-ray microscope in a transmission configuration.

Spectra were recorded at the platinum L_3 edge, using a setup previously described by Nowack et al.[2,7]. The beam, as prepared by the beamline optics was of roughly trapezoidal shape, 1 mm wide and 0.2 mm high. The loaded sample holder was mounted on a movable sample stage with attached heat blower (so that heating is consistent when moving between positions. During the x-ray absorption experiment, at each energy step (step size 1 eV) the sample holder was moved into the beam and 16 images were recorded. Subsequently, the sample holder was moved out of the beam, and another 16 images were recorded as a background. The spectra were obtained by calculating absorbance in each individual point, integrating over the whole image perpendicular to the direction of flow and then integrating in the direction of flow to the desired spatial resolution.

For the operando measurements, the catalyst was exposed to a 1:1 mixture of oxygen and carbon monoxide and heated to 265 °C (light-off temperature at 255 °C). For experiments at reduced oxygen flow, the catalyst was first heated above the light-off temperature before reducing the oxygen flow. The exhaust gas composition was monitored by a Hiden Analytical QGAmass spectrometer.

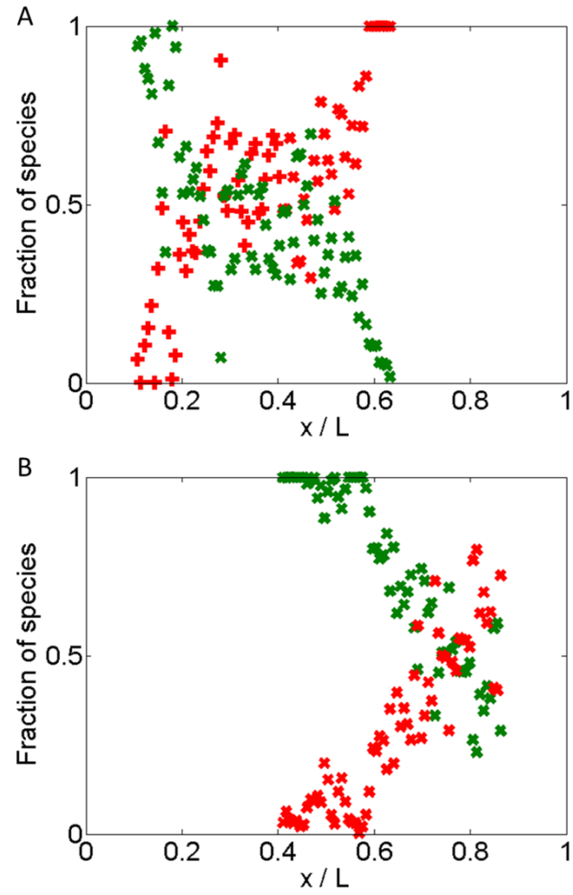

Fig. S5: Amounts of carbon monoxide covered platinum (green) and oxidic platinum (red) along the direction of flow at a carbon monoxide to oxygen ratio of 1:1 (A) and 1:0.8 (B) as determined by linear combination fitting. The transition between the two phases is shifted towards the outlet side with reduced amounts of oxygen.

Figure S5 shows the concentrations of platinum species along the direction of flow under carbon monoxide oxidation conditions. The platinum transitions from a carbon monoxide covered, reduced state near the inlet to oxidized platinum at the outlet. The position of the transition is shifted towards the outlet at higher relative concentrations of carbon monoxide, as is expected from the reactor model derived from the transient experiment.

References

1. Wasserman, S. R. The analysis of mixtures: Application of principal component analysis to XAS spectra. *J Phys IV* **7**, 203-205 (1997).
2. Smolentsev, G. *et al.* Local structure of reaction intermediates probed by time-resolved x-ray absorption near edge structure spectroscopy. *J Chem Phys* **130**, 174508 (2009).
3. Mitchell, M. *An Introduction to Genetic Algorithms* (MIT Press, 1996).
4. MATLAB 7.12 (MathWorks, Natick, MA, 2011).

[5] Szlachetko, J. *et al.* Real Time Determination of the Electronic Structure of Unstable Reaction Intermediates during Au_2O_3 Reduction. *J Phys Chem Lett* **5**, 80-84 (2013).

[6] Alayon, E. M. *et al.* On highly active partially oxidized platinum in carbon monoxide oxidation over supported platinum catalysts. *J Catal* **263**, 228-238 (2009).

[7] Friebel, D. *et al.* In situ X-ray probing reveals fingerprints of surface platinum oxide. *Phys Chem Chem Phys* **13**, 262-266 (2011).
